# Data on growth, productivity, pigments and proximate composition of indigenous marine microalgae isolated from Cox's Bazar Coast

**DOI:** 10.1016/j.dib.2021.106860

**Published:** 2021-02-11

**Authors:** Zahidul Islam, Helena Khatoon, Tashrif Mahmud Minhaz, Mohammad Redwanur Rahman, Shanur Hasan, Yahia Mahmud, Md Shahadat Hossain, Joyshri Sarker

**Affiliations:** a Department of Aquaculture, Chattogram Veterinary and Animal Sciences University, Chattogram 4225, Bangladesh; b Bangladesh Fisheries Research Institute, Mymensingh 2201, Bangladesh

**Keywords:** Marine microalgae, Growth curve, Productivity, Pigments, Proximate composition

## Abstract

Data on growth, productivity, pigments and proximate composition of the four different indigenous marine microalgae (isolated from Cox's Bazar Coast) were collected to compare the growth performance, pigments and nutritional composition. *Chlorella* sp., *Nannochloropsis* sp., *Tetraselmis* sp. and *Chaetoceros* sp. are the four different marine microalgae. Growth curve was determined as the prerequisite to identify the stationary phase for each of the isolated microalgae. Data on growth curves were collected in terms of cell density and optical density to observe the growth rates and division per day. Isolated species were mass cultured in commercial culture medium. When the culture reached at stationary phase, microalgae were extracted to determine productivity, pigments, and proximate composition. The data of productivity (volumetric, areal and lipid productivity), pigments (Chlorophyll a, b, c, carotenoids, and phycobiliproteins), and proximate composition (protein, lipid, and carbohydrate) were significantly (p < 0.05) different among the four different microalgae. Therefore, this data will contribute to the selection of potential microalgae species through proper characterization for vast industrializations.

## Specifications Table

**Table:** 

Subject	Food Science, Aquatic Science
More specific subject area	Microalgae growth, productivity, pigments and proximate composition
Type of data	Table and Chart
How data were acquired	Microscopic and spectrophotometric analysis for growth; biochemical and spectrophotometric analyses for productivity and pigments; biochemical analysis for proximate compositions.
Data format	Raw and analyzed
Parameters for data collection	Conway culture medium was used for algal growth. Four different microalgae were cultured with three replicates. Mass culture was sustained until stationary phase to harvest dried biomass.
Description of data collection	For growth curve: cell density and optical density. For productivity: volumetric productivity, areal productivity and lipid productivity. For pigments: chlorophyll a, b, c, carotenoids, total phycobiliproteins, allophycocyanin, phycocyanin and phycoerythrin. For proximate composition: protein, lipid and carbohydrate.
Data source location	Microalgae lab, Department of Aquaculture, Faculty of Fisheries, Chattogram Veterinary and Animal Sciences University, Khulshi-4225, Chattogram, Bangladesh
Data accessibility	Data are available with this article and also at http://dx.doi.org/10.17632/znns9msdx6.1

## Value of the Data

• This data will contribute to the selection of potential microalgae species through proper characterization on the basis of biomass production, lipid production and pigment accumulation for vast industrializations. This data provides a basis in understanding the profile of four different indigenous marine microalgae species of Bangladesh.
• This data will be useful for the mass production of selected microalgae for specific use. Aquaculture industry can use the information to formulate the feed as well as to enhance the coloration of fishes.
• Growth and productivity data will be useful to plan proper culture method; pigments data will be useful to select species for pigment production. In addition, proximate data will be useful to select potentially commercial species, especially for animal feed and biofuels production.

## 1 Data Description

**Fig. 1:**
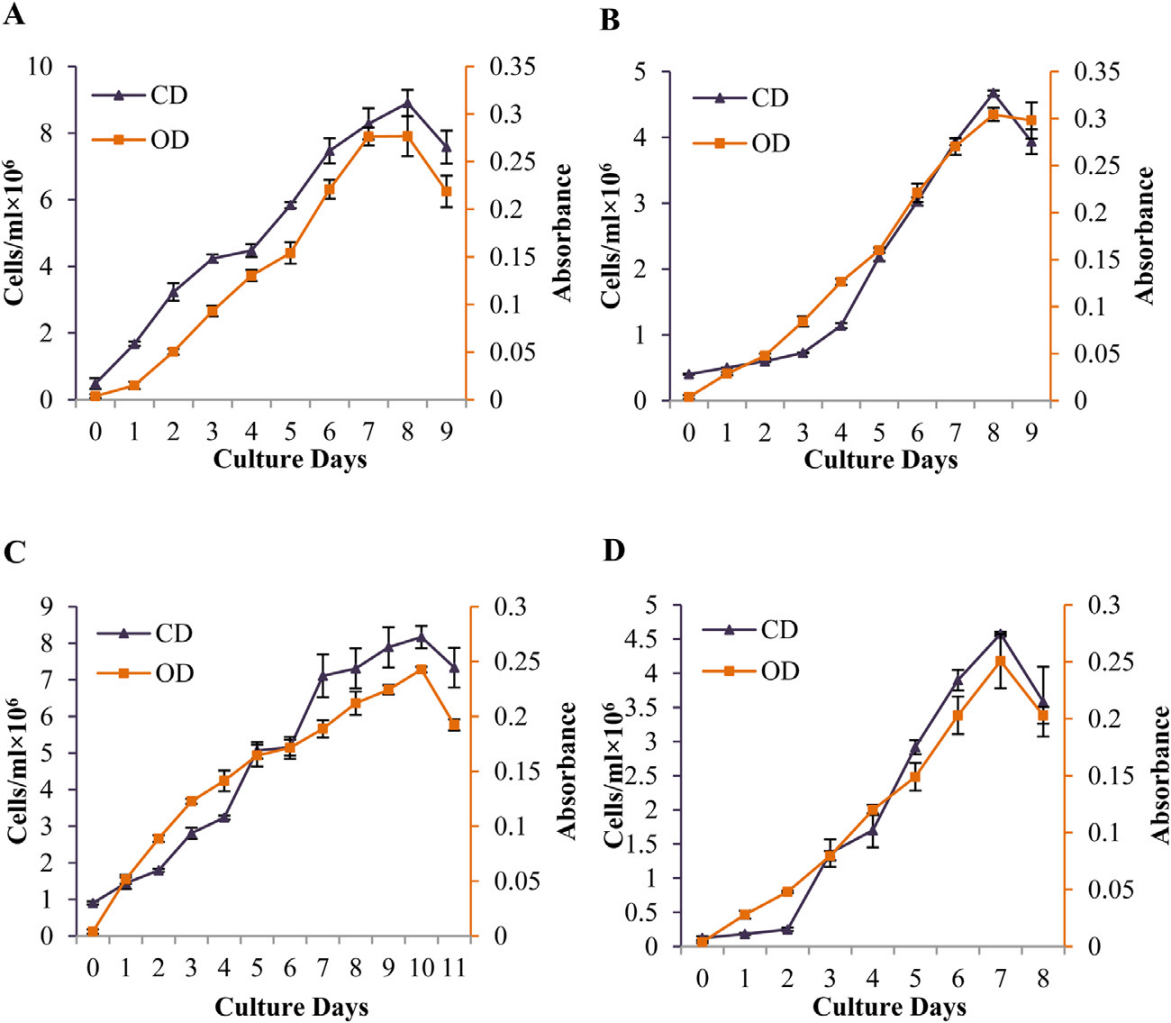
Growth curve in terms of cell density (cells/ml × 10^6^) and optical density (Absorbance) of marine microalgae *Chlorella* sp. (A), *Chaetoceros* sp. (B), *Nannochloropsis* sp. (C), *Tetraselmis* sp. (D). Wavelengths for measuring optical density were 780 nm, 750 nm, 780 nm and 480 nm for A, B, C and D, respectively. Values are means with standard deviation. CD and OD represents cell density, and optical density, respectively.

Growth curves of the four microalgae species, cultured in Conway medium, are shown in this data (Fig. 1). Onset of death phase (7-10 days) varied among the four species.

The volumetric, areal, and lipid productivity of the four microalgae species, cultured in Conway medium, are shown in this data (Table 1). All the data of productivity significantly (p < 0.05) varied among the four different species.

**Table 1:** Volumetric, areal, and lipid productivity of marine microalgae, cultured in Conway medium, and isolated from Cox's Bazar Coast. Values with different letters within each column are significantly different (p < 0.05).

Species	Volumetric productivity (mg/L/day)	Areal productivity (mg/cm^2^/day)	Lipid productivity (mg/L/day)
*Chlorella* sp.	0.39 ± 0.03^c^	0.76 ± 0.06^c^	0.047 ± 0.003^b^
*Chaetoceros* sp.	0.61 ± 0.08^a^	1.21 ± 0.17^a^	0.109 ± 0.003^a^
*Nannochloropsis* sp.	0.45 ± 0.04^bc^	0.89 ± 0.07^bc^	0.108 ± 0.004^a^
*Tetraselmis* sp.	0.57 ± 0.06^ab^	1.12 ± 0.07^ab^	0.051 ± 0.014^b^

Values are means ± SD of triplicate measurements.

**Fig. 2:**
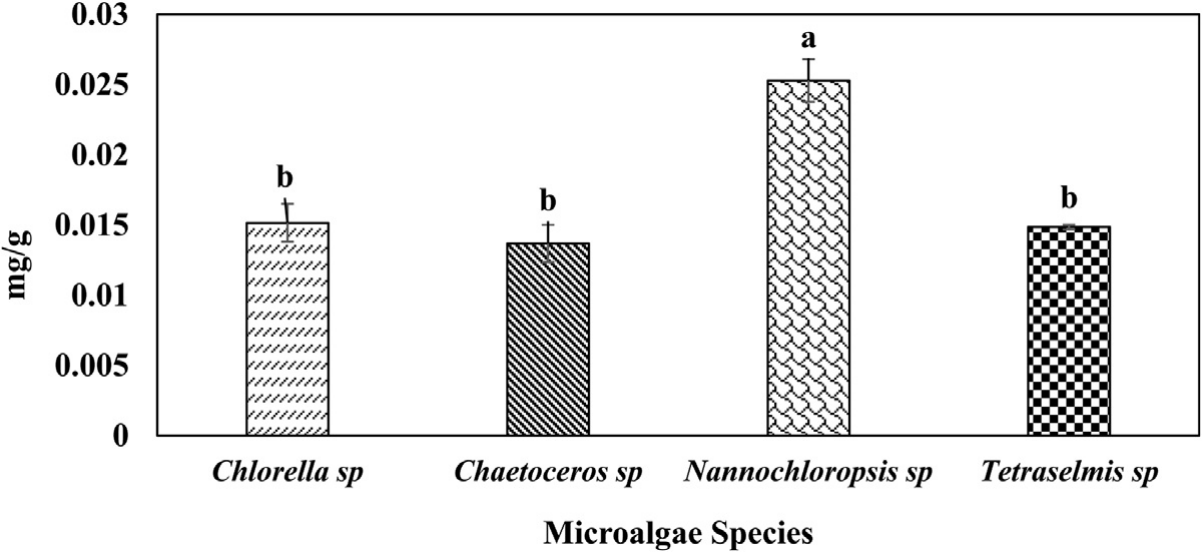
Total phycobiliproteins content (mean ± SD) of marine microalgae, cultured in Conway medium, and isolated from Cox's Bazar Coast. Values with different letters within series are significantly different (p < 0.05).

**Fig. 3:**
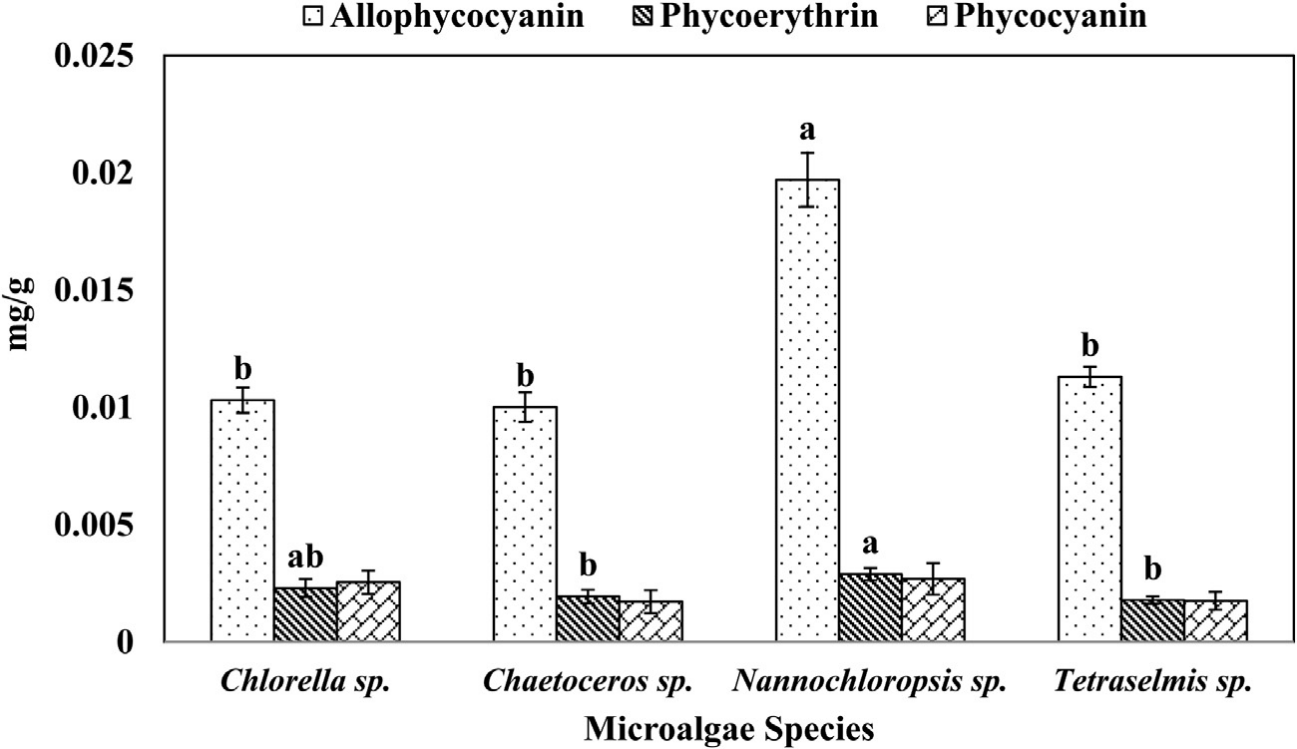
Different phycobiliproteins (mean ± SD) of marine microalgae, cultured in Conway medium, and isolated from Cox's Bazar Coast. Values with different letters within each series are significantly different (p < 0.05).

This data shows chlorophyll a, b, c, carotenoids, and phycobiliproteins (phycocyanin, allophycocyanin and phycoerythrin) production of the four microalgae species, cultured in Conway medium (Table 2, Fig. 2, 3). Table 2 shows that chlorophyll a, chlorophyll b, chlorophyll c, and carotenoids varied significantly (p < 0.05) among the different species. Fig. 2 shows that total phycobiliproteins (0.0137 ± 0.0013 – 0.0253 ± 0.0015 mg/g) varied significantly (p < 0.05) among the four microalgae; similarly, Fig. 3 shows that the three different phycobiliproteins, phycocyanin (0.0017 ± 0.0005 – 0.0027 ± 0.0007 mg/g), allophycocyanin (0.0100 ± 0.0006 – 0.0197 ± 0.0011 mg/g), and phycoerythrin (0.0018 ± 0.0002 – 0.0029 ± 0.0003 mg/g) varied significantly (p < 0.05) among the four different species.

**Table 2:** Chlorophyll a, b, c, and carotenoids content of marine microalgae, cultured in Conway medium, and isolated from Cox's Bazar Coast. Values with different letters within each column are significantly different (p < 0.05).

Species	Chlorophyll a (µg /L)	Chlorophyll b (µg /L)	Chlorophyll c (µg /L)	Carotenoids (µg/mL)
*Chlorella* sp.	0.48 ± 0.05^c^	0.19 ± 0.05^b^	0.06 ± 0.05^bc^	0.56 ± 0.03^b^
*Chaetoceros* sp.	1.30 ± 0.09^b^	0.04 ± 0.02^c^	0.29 ± 0.01^a^	1.36 ± 0.22^a^
*Nannochloropsis* sp.	0.48 ± 0.04^c^	0.05 ± 0.003^c^	0.01 ± 0.01^c^	1.68 ± 0.05^a^
*Tetraselmis* sp.	2.68 ± 0.04^a^	1.23 ± 0.02^a^	0.10 ± 0.01^b^	1.51 ± 0.14^a^

Values are means ± SD of triplicate measurements.

**Fig. 4:**
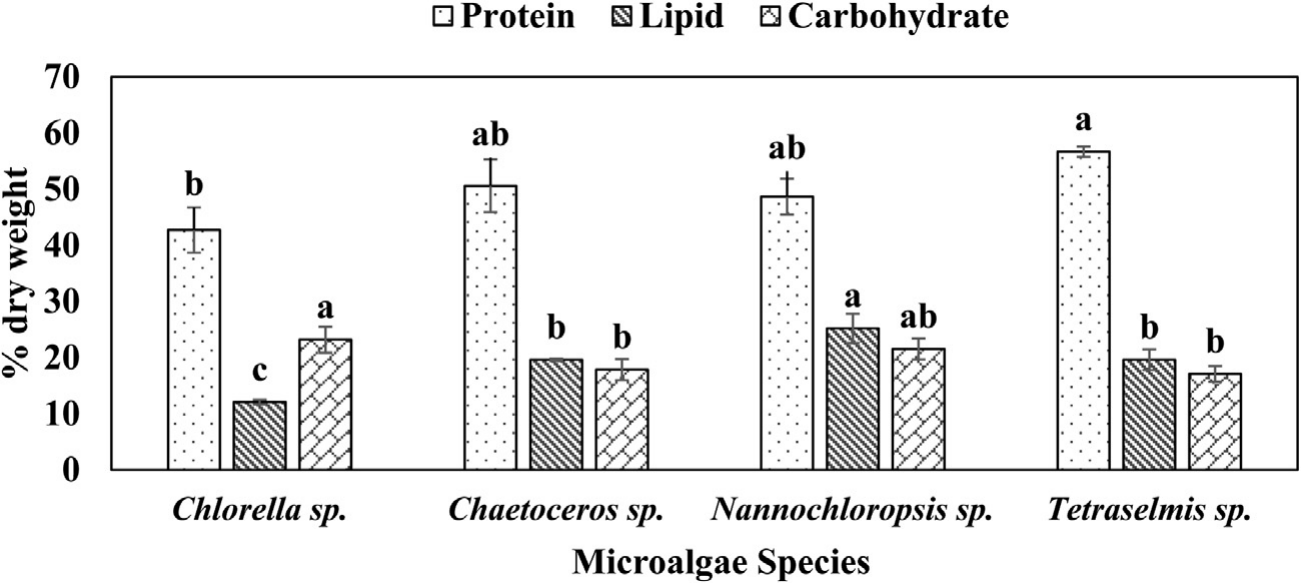
Proximate composition (mean ± SD) of marine microalgae, cultured in Conway medium, and isolated from Cox's Bazar Coast. Values with different letters within each series are significantly different (p < 0.05).

Finally, this data shows protein, lipid and carbohydrate content (% dry weight) of the four different indigenous marine microalgae, culture in Conway medium (Fig. 4). Protein (42.7 ± 4.0 – 56.7 ± 0.9 % dry weight), lipid, (12.1 ± 0.4 – 25.2 ± 2.6 % dry weight) and carbohydrate (17.1 ± 1.4 – 23.2 ± 2.3 % dry weight) data varied significantly (p < 0.05) among the four different species.

## 2 Materials and Method

### 2.1 Media preparation

#### 2.1.1 Filtration, sterilization and preservation of seawater

Seawater was collected from Saint Martin's coast of Bay of Bengal, Bangladesh. After collection, the water was preserved for the settlement of solid particles. Then water was filtered, using 45 mm glass microfiber filters (GF/C). After that, the filtered water was autoclaved at 121°C temperature and 15 lbs. /inch^2^ pressure for 15 minutes. The sterilized water was stored at 20–21^0^C temperature following Reda et al. [1].

#### 2.1.2 Conway medium preparation

Conway medium includes micronutrients, trace metal solution, and vitamin [2]. Pure Conway medium was used for *Chlorella* sp., *Nannochloropsis* sp., *and Tetraselmis* sp. culture*;* however*,* Conway medium + silicate solution was used for *Chaetoceros* sp. culture. Table 3 shows the amount of different constituents. To prepare 1L Conway media, 1 mL of solution A, 0.5 mL of solution B and 0.1 mL of solution C, were added with 28–30 g/L autoclaved seawater (Table 3).

**Table 3:** Constituents of microalgae culture medium.

(A) Main Mineral Solution	
Names of Chemicals	Quantity
NaNOз/KNOз	100.00g/116.00g
Disodium EDTA (C_10_ H_16_N_2_O_8)_	45.00g
H_3_BO_3_	33.60g
NaH_2_PO_4_.4H_2_O	20.00g
FeCL_3_.6H_2_O	1.30g
MnCL_2_.4H_2_O	0.36g
Trace metal solution	1.00mL
Dissolving in deionized/distilled water and make the volume 1 L.	

(B) Trace Metal Solution	
Names of Chemicals	Quantity
ZnCl_2_	2.10g
CoCl_3_.6H_2_O	2.00g
(NH_4_)_6_MO_7_O_2_.4H_2_O	0.90g
CuSO_4_.5H_2_O	2.00g
Dissolving in deionized/distilled water and make the volume 1 L.	

(C) Vitamin	
Names of Chemicals	Quantity
Thiamine, B1	**0.20g**
Cyanocobalamin, B12	**0.01g**
Dissolved in deionized/distilled water and make the volume 100 mL.	

(D) Silicate Solution	
Names of Chemicals	Quantity
Sodium silicate (Na_2_SiO_3_)	**20.00g**
Dissolving in deionized/distilled water and make the volume 1 L.	

#### 2.1.3 Collection of microalgae, culture and maintenance

The four different types of indigenous marine microalgae *(Chlorella* sp*., Nannochloropsis* sp*., Tetraselmis* sp*.,* and *Chaetoceros* sp.*)* were collected from previously preserved samples of the Microalgae laboratory of Department of Aquaculture, Faculty of Fisheries, Chattogram Veterinary and Animal Sciences University, Bangladesh. The pure samples were cultured in Conway culture medium. Stock was scaled up, and then sub cultured for growth curve determination. .

### 2.2 Determination of growth curve

Data of the growth curves were determined prior to the start of the microalgae culture to determine the data of productivity, pigments, and proximate composition. A total of 300 mL of culture volume was maintained in a sterile 500 mL borosilicate Erlenmeyer flask for each species with three replicates. Out of the 300 mL, 270 mL was culture medium and 30 mL was stock culture. The culture was continued up to the death phase. Depending on the species, culture duration was varies which were 9 (*Chlorella* sp.), 11 (*Nannochloropsis* sp.), 9 (*Chaetocersos* sp.) and 8 (*Tetraselmis* sp.) days respectively. Water salinity was maintained 30.1 ± 0.11 g/L, pH was maintained 7.72 ± 0.17, and gentle aeration was provided 24 hrs which offered 4.53 ± 0.53 mg/L dissolved oxygen. Growth curve was determined on basis of cell density (cell/mL), and optical density (absorbance at 780nm for *Chlorella* sp., at 780nm for *Nannochloropsis* sp., at 480nm for *Tetraselmis* sp., and at 750nm for *Chaetoceros* sp.*)*.

#### 2.2.1 Cell density

Microalgal cells were counted using hemacytometer [3] every day during the data collection of growth curve. The hemacytometer and its cover slip (Bright- line improved Neubauer hemacytometer, 0.0025 mm^2^, 0.1 mm deep chambers, Assistent, Germany) were cleaned using Milli- Q water (Millipore Corp.) prior to the fill up of the chambers with culture samples. Evenness of cell distribution was checked under low power magnification (4× and 10×) of the microscope (Nikon E600). Cells were counted for both chambers of the hemacytometer under magnification of 20×; Lugol's iodine was added to culture aliquots for fixation and staining to facilitate counting. The formulae to calculate the cells are as the following:

$$\text{Cell}\;\text{count}\;\text{calculation}\;\left(\text{cell}/\text{ml}\right)\;\text{for}\;5\;\text{squares}\;=\;\frac{\text{Total}\;\text{number}\;\text{of}\;\text{cells}\;\;\text{counted}}{10\;\times \;4}\;\times \;10^{6}$$

Where 10 represented the 10 squares of the 2 hemacytometer chambers and 4 × 10^−6^ represented the volume of samples over the small square areas, that were equivalent to 0.004 mm^3^ (0.2 mm × 0.2 mm × 0.1 mm), expressed in cm^3^ (ml).

$$\text{Cell}\;\text{count}\;\text{calculation}\;\left(\text{cell}/\text{ml}\right)\;\text{for}\;25\;\text{squares}=\frac{\text{Total}\;\text{number}\;\text{of}\;\text{cells}\;\text{counted}}{50\times 4}\times 10^{6}$$

Where 50 represented the 50 squares of the 2 hemacytometer chambers and 4 × 10^−6^ represented the volume of samples over the small square areas, that were equivalent to 0.004 mm^3^ (0.2 mm × 0.2 mm × 0.1 mm), expressed in cm^3^ (mL).

#### 2.2.2 Optical density

Optical denisty of culture aliquots were measured using a spectrophotometer (UV-VIS Double beam, Model-T80, HANNA), every day during the data collection of growth curve. The culture medium for the species was used as the blanks. The absorbance were measured at the wavelength 780nm for *Chlorella* sp., 780nm for *Nannochloropsis* sp., 480nm for *Tetraselmis* sp., and 750 for *Chaetoceros* sp. [4].

### 2.3 Design of the cultures for productivity, pigment, and proximate data

Microalgae were cultured up to stationary phase, in large sterile borosilicate Erlenmeyer flasks with three replicates of each species, thus to collect the data of productivity, pigments and proximate composition. 1.5 L pure Conway medium was taken into each flasks for *Chlorella* sp.*, Nannochloropsis* sp., and *Tetraselmis* sp.; while, 1.5 L Conway medium with silicate solution was taken into each flasks for *Chaetoceros* sp. Then 5% pure culture stock was added to each flask. Each species was cultured separately to inhibit contamination for maintaining pure culture. The culture were conducted at 25.2 ± 0.7°C temperature with 24 hrs light at 150 μ E m^−2^ s^−1^ intensity. Water salinity was maintained 30.1 ± 0.11 g/L, pH was maintained 7.72 ± 0.17, and gentle aeration was provided 24 hrs which offered 4.53 ± 0.53 mg/L dissolved oxygen. Culture was kept in environmental chamber to control the temperature and light throughout the experiment. Culture was maintained until the stationary phase. Culture volume data was recorded every day, while, biomass was determined in every alternate days to determine productivity. For chlorophyll data, 10 mL of each culture was filtered using glass microfiber filter papers (47 mm Ø Whatman® GF/C) at their stationary phase. For Carotenoids data, 1 mL aliquot solution of each culture was collected in 15 mL centrifuge tube. Finally, all the cultures were harvested at their stationary phase based on the growth curve experiment. The cultures were centrifuged to harvest the microalgae (Hitachi* High-speed Refrigerated Centrifuge, himac CR 21g-II). The harvested microalgae was dried at 60°C temperature overnight, using hot air oven, and subsequently, preserved at normal refrigerator (4°C) for pigments and proximate analysis.

### 2.4 Determination of productivity

Volumetric [5], areal [6], and lipid [7] data were determined to estimate productivity. Biomass data is the prerequisite of estimating the productivity data. All the data of productivity was calculated at the stationary phase of different microalgae cultures.

#### 2.4.1 Biomass (Dry Weight Basis)

Biomass was estimated every alternate day using 1 mL microalgae samples from each cultures, filtered through pre-weighed (Rinsed with 1 mL distilled water, and oven dried at 60°C for 4 hrs followed by 1 hr desiccation) glass microfiber filter paper. Then the filter paper with biomass was oven dried again at 60°C for 4 hrs followed by 1 hr desiccation. After that, the dry biomass concentration was calculated by dividing the difference between the weights of the dried filter paper (pre and post filtration) by the filtered volume [3].

#### 2.4.2 Volumetric productivity

Volumetric productivity (VP) indicates the average daily productivity of a culture based on dry weight. Following equation was used to calculate the volumetric productivity:

$$\text{VP}\;(\text{mg}\;L^{-1}\;\text{da}y^{-1})\;=\;\left(X_{n}-X_{0}\right)/N$$

Where, X_n_ = Final biomass, X_0_ = Initial Biomass and N = Culture days

#### 2.4.3 Areal productivity

Areal productivity (AP) is the productivity of an area occupied by the microalgae. Following equation was used to calculate areal productivity:

$$\text{AP}\;(\text{mg}\;cm^{-1}\;\text{da}y^{-1})\;=\;\left(\text{VP}\times V\right)/A$$

Where, VP = Volumetric Productivity, *V* = Total Volume of the culture, A = surface area occupied ground.

#### 2.4.4 Lipid productivity

Lipid productivity (LP) is the amount of lipids produced by microalgae in a day during stationary phase. The lipid productivity was calculated using lipid content and volumetric productivity at the stationary phase. Following equation was used to calculate the lipid productivity:

$$\text{LP}\;(\text{mg}\;L^{-1}\;\text{da}y^{-1})\;=\;\text{VP}\times (\%\;\text{lipid}/100)$$

Where, VP = volumetric productivity of the PBR and, % lipid = lipid content.

### 2.5 Determination of pigments

#### 2.5.1 Extraction of microalgae for chlorophyll determination

For extraction, 10 mL of each sample was filtered (47 mm Ø Whatman® GF/C glass microfiber filter papers). Filter papers with samples were put into airtight plastic bags, and stored frozen (–20°C) for 3 weeks. After 3 weeks, each filter paper with sample was kept submerged in a centrifuge tube with 2–3 mL 90% aqueous acetone solution (Mixing of 90 parts of Acetone with 10 parts of MgCO_3_ Solution), and macerated at 500rpm for 1 min. Then the sample volume was adjusted up to 10 mL with 90% aqueous acetone solution. Subsequently, the samples were steeped for 2 hrs at 4°C temperature. After 2 hrs, the samples were clarified by centrifuging in closed tubes for 20 mins at 500g. Then the clean extract was separated into new tubes.

#### 2.5.2 Determination of chlorophyll

Chlorophyll was determined according to Aminot et al. [8]. The clean extract was transferred to a 1 cm cuvette, and measured optical density (OD) at 750, 664, 647 and 630nm wavelength. OD at 664, 647, and 630nm were used for chlorophyll determination, where OD at 750nm was used as turbidity correction factor. Values of OD at 750nm was subtracted from each of the pigment OD values of the other wavelengths before using them in the equations below:

$$C_{a}\left(\mu g/L\right)=11.85\left(\text{OD}664\right)-1.54\left(\text{OD}647\right)-0.08\left(\text{OD}630\right)$$

$$C_{b}\left(\mu g/L\right)=21.03\left(\text{OD}647\right)-5.43\left(\text{OD}664\right)-2.66\left(\text{OD}630\right)$$

$$C_{c}\left(\mu g/L\right)=24.52\left(\text{OD}630\right)-7.60\left(\text{OD}647\right)-1.67\left(\text{OD}664\right)$$

Where, C_a_, C_b_, and C_c_ = concentrations of chlorophyll a, b, and c, respectively, and OD664, OD647, and OD630 = turbidity corrected optical densities (with a 1-cm light path) at the respective wavelengths.

After determining the concentration of pigment in the extract, following calculation was applied to determine the amount of pigment per unit volume:

$$\text{Chlorophyll}\;\left(\mu g/L\right)=\frac{\text{Chlorophyll}\;a\times \text{Extract}\;\text{Volume}\;\;\text{in}\;\;\text{mL}}{\text{Volume}\;\text{of}\;\text{sample}\;\;\text{in}\;L}$$

#### 2.5.3 Determination of carotenoids

1 mL aliquot of the algal suspension of each culture were taken at their stationary phase, and centrifuged at 1000g for 5 mins to obtain pellet. Afterwards, the pellet was extracted with 3 mL 2:1 of ethanol:hexane (v/v).Then the pellet with the solvent was shaken vigorously, and centrifuged again at 1000g for 5 mins. Thus, the hexane layer was separated, and its absorbance was determined spectrophotometrically at the wavelength of 450nm. The amount of extracted carotenoids from the samples in micrograms was determined by multiplying the absorbance (A_450_) with 25.2 [9].

#### 2.5.4 Extraction of phycobiliproteins

The cultures were centrifuged at 6,000 rpm at room temperature for 15 mins to harvest the pellet. The cell pellets were rinsed 2–3 times with distilled water. The harvested biomass was dried in oven at 40°C overnight. Dried powder (40 mg) was then soaked in 10 mL phosphate buffer (pH 7.0; 0.1 M), mixed well using vortex mixture, and then stored at 4°C for 24 hrs. Phycobiliproteins were extracted by centrifuging at 6000 rpm for 10 mins. Finally the supernatant was collected and absorbance was measured spectrophotometrically (UV-VIS Double beam, Model-T80, HANNA) at the wavelength 562, 615, and 652nm; phosphate buffer was used as blank.

#### 2.5.5 Spectrophotometric estimation of phycobiliproteins

The amount of phycocyanin (PC), phycoerythrin (PE) and allophycocyanin (APC) in the sample was calculated from the absorbance using the following equations and the extinction coefficients from Siegelman and Kycia [10]:

$$\text{PC}\;\left(\text{mg}/\text{mL}\right)=\frac{A_{615}-\left(0.474\;\times A_{652}\right)}{5.34}$$

$$\text{APC}\;\left(\text{mg}/\text{mL}\right)=\frac{A_{652}-\left(0.208\;\times A_{615}\right)}{5.09}$$

$$\text{PE}\;\left(\text{mg}/\text{mL}\right)=\frac{A_{562}-\left(2.41\;\times \;\text{PC}\right)-\left(0.849\;\times \;\text{APC}\right)}{9.62}$$

Total phycocyanin, phycoerythrin, and allophycocyanin (mg/g) were calculated according to Silveira et al. [11] as follows:

P=(PigmentconcentrationV)/DB

Where, V= solvent volume, DB= Dried biomass

Total phycobiliproteins (mg/g) were further calculated from the sum of the phycocyanin, phycoerythrin, and allophycocyanin contents in dried microalgae biomass.

### 2.6 Determination of proximate compositions

#### 2.6.1 Carbohydrate determination

Carbohydrate was determined according to Dubois et al. [12]. For each sample, 5 mg freeze dried biomass was taken to prepare a 25 mL well mixed (tissue homogenizer) solution using distilled water. Afterwards, 1 mL from 25 mL solution was taken for each samples, and then 1 mL of 5% phenol solution and 5 mL of sulfuric acid were added into it. Then, the samples were kept in a cold water bath. When cooled, absorbance of the solution was taken at 488nm wavelength using spectrophotometer to estimate carbohydrate. To produce a calibration graph, 1000 µg/L of standard (glucose) stock solution was prepared, and subsequently, a series of standards at various dilution (20 µg /L, 40 µg /L, 60 µg /L, 100 µg /L and 140 µg /L) were also prepared from the stock solution. The same procedures as described for carbohydrate analysis were applied for the standard series; a standard graph was plotted according to the standard results obtained from the absorbance, and the carbohydrate composition for every sample was determined accordingly.

#### 2.6.2 Protein determination

Protein was determined according to Lowry et al. [13]. For each sample, 5 mg of freeze dried microalgae biomass was taken to prepare a 25 mL well mixed (tissue homogenizer) solution using distilled water. 0.5 mL from 25 mL solution was taken for each samples for protein analysis. 50 mL of Reactive 2 (2g of Na_2_CO3 in 100 mL of 0.1 NaOH) and 1 mL of Reactive 1 (1% NP tartrate) were mixed. After that, 0.5 mL sample was added with 0.5 mL of 1N NaOH, and it was kept in a hot water bath at 100°C for 5 mins. Subsequently, the samples were cooled in a cold water bath, and 2.5 mL of the prepared mixed reagent was added 10 mins after cooling. After that, 0.5 mL of Falin reagent was added to the mixed reagent, and then kept in a dark places for 30 mins. The absorbance of the mixed solution was measured using spectrophotometer at 750 nm wavelength. To develop a calibration graph, 2000µg/L of standard (albumin) stock solution was prepared, and a series of standards were prepared (20 µg/L, 40 µg/L, 80 µg/L, 100 µg/L and 200 µg/L) from the stock solution. The same procedures as described for protein analysis were applied for the standard series; a calibration line was plotted according to the absorbance, and the protein composition for each sample was determined accordingly.

#### 2.6.3 Lipid determination

Lipid was determined according to Bligh and Dyer [14], and Folch et al. [15]. For each sample, an aluminum dishes were labeled and weighted as initial weight. Then 50 mg of each sample was taken in a centrifuge tube, and diluted into 5x volume using distilled water. Then, 3 mL 2:1 of methanol:chloroform (v/v) was mixed with the sample homogenously using tissue homogenizer. After that, all the tubes were centrifuged for 4mins at 1000 rpm at 4°C; the supernatants were transferred into clean tubes by Pasteur pipette, and placed them in ice. Again, 3 mL 2:1 of methanol:chloroform (v/v) was mixed with the sample homogenously. After that, the tubes were centrifuged at same conditions again, and supernatants were transferred to the previous tubes of supernatants. In this combined supernatant, 1.5 mL of 0.9% NaCl was mixed using vortex mixture. Then the tubes were kept in the refrigerator for 1 hr at 4°C temperature. After 1 hr, the tubes were centrifuged for 10 mins at 1000 rpm at 4°C temperature. The upper layer of methanol and chloroform was discarded, while, the lower layer was transferred in previously made aluminum dish. The solvent was then evaporated at 60°C by hot air oven. Afterwards, the aluminum dishes were weighed to get the final weight. Finally, initial weight was subtracted from the final weight to get the lipid weight in the samples.

### 2.7 Statistical analysis

Mean and standard deviation of mean were calculated using MS excel. When assumptions were met, ANOVA was applied to test the significance of the difference of productivity, pigments, and proximate composition among the four different microalgae. IBM SPSS (v. 26.0) was used for ANOVA.

## Ethical Statement

These data were collected complying ARRIVE guidelines. Ethical approval is not a prerequisite of starting the data collection procedure for microalgae.

## Declaration of Competing Interest

The authors disclose that they have no known conflict of interests that may have influenced either the data collection or the presentation of the data.
